# Evaluation of Multiplex PCR with Enhanced Spore Germination for Detection of *Clostridium difficile* from Stool Samples of the Hospitalized Patients

**DOI:** 10.1155/2013/875437

**Published:** 2013-03-17

**Authors:** Surang Chankhamhaengdecha, Piyapong Hadpanus, Amornrat Aroonnual, Puriya Ngamwongsatit, Darunee Chotiprasitsakul, Piriyaporn Chongtrakool, Tavan Janvilisri

**Affiliations:** ^1^Department of Biology, Faculty of Science, Mahidol University, Bangkok 10400, Thailand; ^2^Department of Tropical Nutrition and Food Science, Faculty of Tropical Medicine, Mahidol University, Bangkok 10400, Thailand; ^3^Department of Clinical Sciences and Public Health, Faculty of Veterinary Science, Mahidol University, Nakhon Pathom 73170, Thailand; ^4^Department of Medicine, Faculty of Medicine Ramathibodi Hospital, Mahidol University, Bangkok 10400, Thailand; ^5^Department of Pathology, Faculty of Medicine Ramathibodi Hospital, Mahidol University, Bangkok 10400, Thailand; ^6^Department of Biochemistry, Faculty of Science, Mahidol University, Bangkok 10400, Thailand

## Abstract

*Clostridium difficile* poses as the most common etiologic agent of nosocomial diarrhea. Although there are many diagnostic methods to detect *C. difficile* directly from stool samples, the nucleic acid-based approach has been largely performed in several laboratories due to its high sensitivity and specificity as well as rapid turnaround time. In this study, a multiplex PCR was newly designed with recent accumulated nucleotide sequences. The PCR testing with various *C. difficile* ribotypes, other *Clostridium* spp., and non-*Clostridium* strains revealed 100% specificity with the ability to detect as low as ~22 genomic copy number per PCR reaction. Different combinations of sample processing were evaluated prior to multiplex PCR for the detection of *C. difficile* in fecal samples from hospitalized patients. The most optimal condition was the non-selective enrichment at 37°C for 1 h in brain heart infusion broth supplemented with taurocholate, followed by the multiplex PCR. The detection limit after sample processing was shown as being 5 spores per gram of fecal sample. Two hundred and thirty-eight fecal samples collected from the University affiliated hospital were analyzed by the enrichment multiplex PCR procedure. The results suggested that the combination of sample processing with the high-performance detection method would be applicable for routine diagnostic use in clinical setting.

## 1. Introduction

*Clostridium difficile* is a motile, rod-shaped, Gram-positive bacterium, which is known to be a leading cause of antibiotic-associated diarrhea, especially nosocomial infections [1]. Though *C. difficile* is not a major component of natural gut flora, treatment with broad-spectrum antibiotics impedes the growth of other bacterial species and allows *C. difficile* to colonize. Following the colonization, an enterotoxin, TcdA, which is found in ~70% of *C. difficile* strains, and a cytotoxin, TcdB, which is found in all *C. difficile* strains, can be produced, thereby disrupting tight junctions of the intestinal epithelial cells resulting in inflammation and increased permeability of the intestine [2]. Approximately less than 10% of clinical *C. difficile* isolates possess binary toxins (cdt*A/B*), which have been associated with increased severity of the symptoms [3]. The pathogenic role of cdt*A/B* has been suggested to trigger microtubule protrusion, thereby increasing the adherence of *C. difficile* to the gut epithelium [4]. *C. difficile* infection (CDI) results in a wide range of symptoms including fever, abdominal pains, mild diarrhea, and pseudomembranous colitis. Although CDI can be treated with certain antibiotics, the emergence of hypervirulent strains that are resistant to current chemotherapy and are able to produce high titers of toxins poses a challenge to the treatment of CDI worldwide [5].

To date, there are several diagnostic assays for the detection of *C. difficile*, each of which reveals the advantages and disadvantages and discrepancies in the performance existing in the literature [6, 7]. Conventional diagnostic methods, including toxigenic bacterial cell culture and tissue cell culture cytotoxicity neutralization assays, have been considered as the reference standard [8]. These assays require technical expertise and several days to obtain results: therefore, they are not appropriate for the clinical setting, where an accurate and rapid diagnosis is needed. An enzyme immunoassay (EIA) for TcdB alone or both TcdA and TcdB has been widely used in most laboratories because it is relatively simple, rapid, and commercially available [9]. However, it has been revealed that the sensitivity of EIA is as low as 23% and the specificity as low as 75% [10]. Therefore, in practice, a symptomatic patient with the EIA-negative result is usually tested by another assay with higher sensitivity. Many laboratories have reported the combination of assays to increase the sensitivity and specificity of the detection [11–13]. An example include a 2-step algorithm, in which the first step is to perform an EIA for glutamate dehydrogenase (GDH) and the second step is to test the GDH-positives with an EIA for toxins. The EIA for GDH step yields a highly sensitive result for *C. difficile*, but is not specific for toxigenic isolates; therefore, the GDH-positive results must be confirmed with a subsequent specific test such as EIA for toxins [13]. Although these algorithms improve the diagnostic performance, it has been shown that the levels of sensitivity of the EIA for GDH in this two-step algorithm vary depending on the *C. difficile* ribotypes [14]. Moreover, the two- or more step assays are cost-ineffective [15]. Recently, nucleic acid amplification tests (NAATs) have been developed as a single assay with the same day results for CDI. These assays aim to detect the toxin gene(s) and have been proven to be more superior than other methods, except the toxigenic bacterial cell culture, as they yield the high sensitivity and high negative predictive value [16]. Currently, there are a number of FDA-approved commercially available NAATs including (i) the Xpert *C. difficile*, (ii) Xpert *C. difficile*/Epi assays that detect *tcdB* by real-time PCR, and (iii) the Illumigene *C. difficile *assay that detects *tcdA* by loop-mediated isothermal amplification [17].

Although the NAATs have gained popularity for CDI diagnosis, the common drawbacks of this type of assays to detect pathogens directly from stool samples are the presence of PCR inhibitors, contamination of DNA from host and other microorganisms, and low quality and yield of bacterial DNA that is extracted from spores in stool samples from suspected patients. Thus, the objective of this study was to evaluate the multiplex PCR with enhanced spore germination for the detection of *C. difficile* directly from stool samples of hospitalized patients. The combination of sample processing with the high-performance detection method would be applicable for routine diagnostic use in clinical setting.

## 2. Materials and Methods

### 2.1. Specimen Collection and Acquisition

A total of 238 fecal specimens from inpatients that aged more than 15 years and developed diarrhea during hospitalization at Ramathibodi hospital, a 1,000-bed tertiary health care university Hospital, were collected from May 2010 to January 2011. The samples were subjected to the routine EIA test using VIDAS *C. difficile* Toxin A&B qualitative assay (BioMérieux, Marcy l'Etoile, France) according to the manufacturer's recommendations. The samples were also subjected to *C. difficile* selective culture by plating onto cycloserine cefoxitin fructose agar (CCFA) and incubated anaerobically at 37°C for up to 5 days. All samples were then subsequently stored at −80°C before use. The use of human materials has been approved by the research ethics committee of the Faculty of Medicine at Ramathibodi Hospital, Mahidol University, Thailand.

### 2.2. Bacterial Cell Culture

*Clostridium* strains were grown anaerobically in BHIS medium, brain heart infusion broth at 37°C (Oxoid, Basingstoke, UK), supplemented with 5% yeast extract, 0.1% sodium thioglycolate (TCI, Tokyo, Japan), and 0.1% L-cysteine (TCI). Before sterilization, anaerobic conditions were created by boiling the medium for 10 min and, during cooling, flushing the medium with nitrogen gas. All other bacteria were cultivated at 37°C in tryptone soy broth (Oxoid).

### 2.3. Multiplex PCR for the Detection of *C. difficile* Toxin Genes

A multiplex PCR was developed for the detection of toxin genes including *tcdA*, *tcdB*, *cdtA*, and *cdtB* as well as *16S rDNA* as a internal control. A total volume of 20 *μ*L PCR reaction consisted of 1 × PCR buffer (10 mM Tris-HCl, 50 mM KCl, and pH 8.3), 5 mM MgCl_2_, 250 *μ*M dNTP, 1 × enhancer (0.5 M betaine, 1% DMSO), 1 U of Taq DNA polymerase (New England Biolabs, MA, USA), and 5 pairs of primers at indicated concentrations (Table 1). Thermal cycling parameters included (i) an initial denaturation at 92.5°C for 2 min; (ii) 30 cycles of denaturation at 92.5°C for 20 s, annealing at 60°C for 65 s, and extension at 68°C for 70 s; and (iii) a final extension at 68°C for 5 min. PCR products were resolved by electrophoresis on a 2% agarose gel stained with ethidium bromide.

### 2.4. Preparation of *C. difficile* Spores

Spores from *C. difficile* strain R20291 were produced in a sporulation medium as described previously [18]. Briefly, a culture of *C. difficile* was spread onto BHIS agar supplemented with 0.1% taurocholate (BHIS/TA). The plates were then incubated at 37°C under anaerobic conditions for 5 days. Spores were scraped off the plates and resuspended in deionized water. The samples were then washed ten times with water. The spores were checked for purity and enumerated using phase-contrast microscopy and light microscopy after staining with malachite green and eosin Y. Spore samples were then stored at −20°C until further analysis.

### 2.5. Enrichment PCR Procedures

In order to evaluate the effects of enhanced spore germination and enrichment on the multiplex PCR detection of *C. difficile*, two consecutive methods were performed with spore-inoculated feces prior to the multiplex PCR. One hundred *C. difficile* spores were spiked into 1 gram of homogenized fecal samples. Bulk debris was avoided during sample withdrawal. The spiked samples were then subjected to the pretreatment conditions with or without alcohol shock for 20 min. Alcohol shock should eliminate vegetative bacterial cells from the samples, leaving viable spores to germinate. Following the alcohol shock, either nonselective spore germination medium BHIS/TA alone or selective BHIS/TA/CC medium (BHIS/TA in the presence of 250 mg/L cycloserine and 20 mg/L cefoxitin) was added to the samples, which were incubated anaerobically at 0, 1, 2, and 3 h. The samples were then divided to two halves, one of which was subjected to bacterial DNA extraction using EZNA stool DNA kit (Omega, GA, USA); the other was then cultured on either BHIS or CCFA plates. All experiments were performed in triplicate.

## 3. Results and Discussion

Toxigenic *C. difficile* strains are recognized as the main cause of nosocomial diarrhea [1–3]. Therefore, a rapid and cost-effective method to detect *C. difficile* directly from stool samples facilitates patient management to control CDI. The aim of this work was to design an optimized multiplex PCR for the detection of toxigenic *C. difficile* from stool samples of hospitalized patients and to evaluate the combination of various sample processing conditions and multiplex PCR on such detection.

### 3.1. Multiplex PCR for the Detection of Toxigenic *C. difficile*

**Figure 1:**
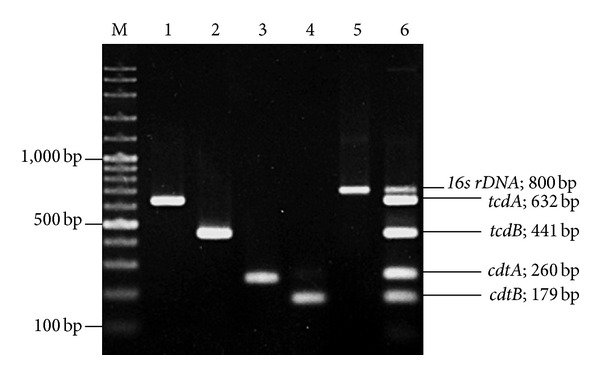
Agarose gel electrophoresis of the PCR products tested with five primer pairs using 20 ng of genomic DNA of *C. difficile* R20291 as template. Each primer pair was tested individually and in combination with all 5 primer pairs in the multiplex PCR. Lane M: 100-bp DNA ladder marker. Lanes 1–5, single-plex PCR reactions using primers specific to *tcdA*, *tcdB*, *cdtA*, *cdtB*, and *16s rDNA*, respectively. Lane 6: multiplex PCR with all four toxin-specific primers and 16S rDNA primers.

**Figure 2:**
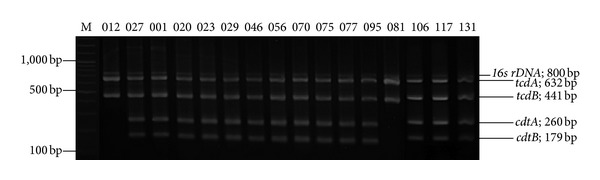
Multiplex PCR toxin gene amplification profiles of various *C. difficile* ribotypes. Lane M: 100 bp DNA ladder marker. The ribotypes and PCR products of detected genes are indicated.

In the past years, there have been an increasing number of *C. difficile* genome and toxin gene sequences deposited in the GenBank database, enabling us to design more specific primers. The 5-plex PCR primers were developed for the detection of the four *C. difficile* toxin genes including *tcdA*, *tcdB*, *cdtA*, and *cdtB*, together with *16S rDNA* as an internal PCR control (Figure 1). The primer set was chosen to amplify products with distinguishable sizes on agarose gel electrophoresis. The individual primer pairs for the amplification of the regions in the *tcdA*, *tcdB*, *cdtA* and *cdtB* genes were tested in single-plex PCRs (Figure 1; lanes 1–4, respectively). The multiplex PCR with a combination of all five primer pairs was optimized (Figure 1; lane 5) and was tested with different PCR *C. difficile* ribotypes (Figure 2). Our results are consistent with the previously reported data [19].

### 3.2. Sensitivity and Specificity Test

The sensitivity and specificity of the developed multiplex PCR for the detection of *C. difficile* were evaluated. The detection limit as measured with genomic DNA from toxigenic reference strain *C. difficile* R20291 was 0.1 pg or ~22 genomic copy number per reaction. To further evaluate the primer specificities for *C. difficile*, 7 other* Clostridium* spp. strains including *C. septicum*, *C. glycolicum*, *C. perfringens*, *C. tetani*, *C. sordellii*, *C. sporogenes*, and *C. botulinum*, as well as 7 of non-*Clostridium* strains including *Bacillus cereus*, *Salmonella* Typhi, *Shigella dysenteriae*, *Escherichia coli*, *Enterobacter faecalis*, *Klebsiella ozaenae*, and *Citrobacter fundi* were tested. None of the non-*C. difficile* strains gave rise to the PCR-positive results, thereby indicating the specificity of the multiplex PCR assay. 

### 3.3. Enrichment Multiplex PCR for Detection of Toxigenic *C. difficile*

The multiplex PCR assays for detection of toxigenic *C. difficile* directly from fecal specimens have been previously reported [20–22]. However, the detection limit has been shown to be as low as 5 × 10^4^ cfu per 1 g of feces [22]. The sensitivity of the PCR is usually affected by inhibitors found in stool samples including bile salts, complex polysaccharides, proteinases, a high concentration of background flora, and a low concentration and uneven distribution of target microorganism [23], rendering it not suitable for PCR detection of the pathogen from direct stool samples. In this study, different combinations of pretreatment conditions, enrichment conditions, and enrichment times prior to both multiplex PCR and conventional bacterial cell culture methods were evaluated to determine the possibility of detecting 100 *C. difficile* spores per 1 g of feces (Table 2). This low spore concentration was not detected by nonenrichment multiplex PCR and culture methods. Regarding the pretreatment step, the *C. difficile* colonies were observed on CCFA after 1 h of enrichment without alcohol shock, compared to 3 h with the alcohol shock. Under nonselective plating, most conditions yielded uninterpretable results due to the high levels of contamination of other microorganisms. Positive PCR results were obtained under the conditions of the 1 h enrichment time, regardless of pretreatment or enrichment broth. Therefore, the condition of no alcohol shock and enrichment with BHIS/TA for 1 h was identified as the optimal combination with practical working hours in laboratories and was appropriate for both bacterial cell culture and multiplex PCR diagnosis. Furthermore, the sensitivity of the developed multiplex PCR under the described conditions was also tested with fecal samples spiked with different numbers of spores. We found that the multiplex PCR was able to detect as low as 5 spores/g feces. Therefore, the enrichment broth could enhance spore germination and transformation into vegetative cells, allowing them to grow to PCR-detectable levels. 

### 3.4. Detection of Toxigenic *C. difficile* in Clinical Stool Samples

To examine the efficacy of the assay described in this study, 238 stool samples of patients suspected of CDI were subjected to multiple PCR with the enhanced spore germination as above. Routine EIA and bacterial cell culture assays were also performed. All samples were tested positive for the *16S rDNA* control gene. Comparison of different diagnostic assays is shown in Table 3. Sixteen cases were tested positive while 166 cases gave rise to negative results in all three assays. Eighteen samples were EIA positive, but both PCR and culture negative, and were, therefore, considered to be negative due to the low level of EIA specificity that requires the second assay to confirm the EIA results. Twenty-four cases were shown to be PCR positive with either EIA positive or culture positive. These results represented another two-step diagnostic process that increased the reliability of the PCR outcomes. Twenty-four samples were PCR positive, but both EIA and culture turned out negative. Although anaerobic culture was considered the most sensitive assay for the detection of *C. difficile*, it is also likely that the low spore counts in fecal samples and different rates of spore viability would account for such results. However, another confirmatory assay should be performed to support the PCR results. Of 54 PCR-positive samples, 7 were *tcdA* ^+^ *tcdB* ^+^ *cdtA*/*B* ^+^, 13 were *tcdA* ^+^ *tcdB* ^+^ *cdtA*/*B* ^−^, 30 were *tcdA* ^−^ *tcdB* ^+^ *cdtA*/*B* ^−^, and 4 were *tcdA* ^−^ *tcdB* ^+^ *cdtA*/*B* ^−^. Although, there are previous reports on *C. difficile* toxin types in Thailand [24, 25], this is the first time that the *tcdA* ^+^ *tcdB* ^+^ *cdtA*/*B* ^+^ isolates were detected. It is also noteworthy that PCR detection of *C. difficile* toxin genes might only detect DNA without the presence of toxin production; therefore, the PCR-positive cases should be carefully interpreted.

Although the enrichment multiplex PCR in this study showed superiority over the other two diagnostic methods, the limitations of our experimental setting include the lack of another confirmatory diagnostic test such as cell cytotoxicity; so the false positives or negatives in cases of discordant test results could not be identified. However, analytical sensitivity with the reference *C. difficile* strain and spore-inoculated fecal samples and specificity of the multiplex PCR assay with various *C. difficile* ribotypes, other *Clostridium* spp., and other bacteria species revealed high sensitivity with no false positive or false negative. 

## 4. Conclusions

This work revealed the comprehensive evaluation of sample processing prior to the multiplex PCR diagnosis of *C. difficile* directly from clinical stool samples. The results demonstrated that the enrichment of nonselective medium for 1 h prior to both PCR or bacterial cell culture assays yielded the most optimal condition. The performance of the enrichment multiplex PCR was proven to be more superior to that of the routine EIA. This rapid and cost-effective diagnostic assay provides an alternative approach for the detection of *C. difficile*, thereby improving the CDI management. However, a large-scale clinical testing is warranted to further validate this assay. 

## Figures and Tables

**Table 1:** Primers in the multiplex PCR for the detection of *C. difficile. *

Target	Primer	Sequence (5′-3′)	Primer concentration (*μ*M)	Amplicon size (bp)
*tcdA *	tcdA-F	GTATGGATAGGTGGAGAAGTCAGTG	0.025	
	tcdA-R	CGGTCTAGTCCAATAGAGCTAGGTC	0.025	632
*tcdB *	tcdB-F	GAAGATTTAGGAAATGAAGAAGGTGA	0.01	
	tcdB-R	AACCACTATATTCAACTGCTTGTCC	0.01	441
*cdtA *	cdtA-F	ATGCACAAGACTTACAAAGCTATAGTG	0.2	
	cdtA-R	CGAGAATTTGCTTCTATTTGATAATC	0.2	260
*cdtB *	cdtB-F	ATTGGCAATAATCTATCTCCTGGA	0.5	
	cdtB-R	CCAAAATTTCCACTTACTTGTGTTG	0.5	179
*16s rDNA *	UFU-L	GCCTAACACATGCAAGTCGA	0.025	
	UR802	TACCAGGGTATCTAATCC	0.025	800

**Table 2:** Evaluation of pretreatment and enrichment conditions prior to multiplex PCR and bacterial cell culture for the detection of *C. difficile* directly from stool samples.

Pretreatment condition	Enrichment condition	Incubation time (h)	Typical *C. difficile* colony		Multiplex PCR toxin genes detection
			CCFA agar plate	BHI agar plate	
Alcohol shock	BHIS/TA	0	−	−	−
		1	−	−	+
		2	−	ND	+
		3	+	ND	+
	BHIS/TA/CC	0	−	−	−
		1	−	−	+
		2	−	ND	+
		3	+	ND	+

No alcohol shock	BHIS/TA	0	−	ND	−
		1	−/+	ND	+
		2	+	ND	+
		3	+	ND	+
	BHIS/TA/CC	0	−	ND	−
		1	−/+	ND	+
		2	+	ND	+
		3	+	ND	+

ND stands for “not determined” because there were too many contaminated bacterial species, rendering it impossible to distinguish *C. difficile* colonies on the plates. −/+ indicates that typical *C. difficile* colonies could not be observed in at least one of the three replicates.

**Table 3:** Comparison of different diagnostic assays including toxin EIA, multiplex PCR, and bacterial cell culture.

Toxin EIA	Multiplex PCR	Bacterial cell culture	Number of cases
+	+	+	16
+	+	−	4
+	−	−	18
−	+	+	10
−	+	−	24
−	−	−	166
